# Co-profiling of translatome and transcriptome reveals the regulation of dynamic gene expression during *Drosophila* embryogenesis: Co-profiling of translatome and transcriptome during *Drosophila* embryogenesis

**DOI:** 10.3724/abbs.2024146

**Published:** 2024-09-02

**Authors:** Le Zhang, Qiufang Liu, Yulong Liu, Bishan Ye, Chuansheng Hu, Xinhui Li, Ling Bai, Ming Cheng, Mingzhu Zhao, Hongmei Li, Hua Li

**Affiliations:** 1 Jiangsu Province Engineering Research Center of Molecular Target Therapy and Companion Diagnostics in Oncology Suzhou Vocational Health College Suzhou 215009 China; 2 Bio-ID Center School of Biomedical Engineering Shanghai Jiao Tong University Shanghai 200240 China; 3 Health School Attached to Shanghai University of Medicine & Health Sciences Shanghai 200237 China; 4 School of Agriculture and Biology Shanghai Jiao Tong University Shanghai 200240 China; 5 Instrumental Analysis Center Shanghai Jiao Tong University Shanghai 200240 China; 6 Department of Cardiology and Shanghai Institute of Precision Medicine Ninth People’s Hospital Shanghai Jiao Tong University School of Medicine Shanghai 200125 China

Eukaryotic gene expression is regulated at multiple levels, aiding in maintaining normal phenotypes and environmental adaptability. Transcriptional regulation complexity has been extensively studied using high-throughput sequencing, and previous studies have shown that different transcript isoforms can be produced through complex regulatory mechanisms via large-scale RNA sequencing. Additionally, translational regulation, which significantly influences gene expression, is controlled by complex mechanisms [1]. The untranslated regions (UTRs) of eukaryotic mRNA, encompassing the 5′ UTR, 3′ UTR and polyadenylation tail (polyA), are pivotal for translational regulation, with distinct cis-regulatory elements in the 5′ UTR and 3′ UTR of various transcript isoforms, leading to substantial variations in translational regulation across transcripts. To shed light on translational regulation, previous studies have performed isolation of ribosome-associated poly-adenylated RNAs ( *i*. *e*., translatome) and deep sequencing for mRNA translation [2]. Polysome profiling is the most common method used to study translatome, which can enable the isolation of full-length translated mRNAs, thereby facilitating the identification of UTRs, assessment of selective translation, and comprehension of the regulatory mechanisms underlying gene expression [2].

*Drosophila* embryonic development progresses very rapidly and requires precise regulation of the transcription and translation of a large number of genes to ensure normal gene expression. Although *Drosophila* has been extensively studied as a model organism, the specific interplay between transcription and translation during embryonic development stages is not yet fully understood. To investigate the dynamic regulation of gene expression during *Drosophila* embryogenesis, we conducted transcriptome and translatome co-profiling on early (0‒4 h) embryos and S2R+ cells, a cell line derived from late emb ryonic stages of *Drosophila melanogaster* [3], to compare the differences in translational regulation at the gene and transcript isoform levels.

S2R+ cell culture and early (0–4 h) embryo collection were performed (see Suppleme ntary Methods) to compare transcriptome and translatome profiling, as shown in Supplementary Figure S1. Cytosolic RNA and ribosome-associated RNA were isolated from embryos [4] and S2R+ cells, which were used for constructing RNA-Seq libraries. Four libraries were generated for RNA-seq (see Supplementary Methods), consisting of two cytosolic RNA libraries and two ribosome-associated RNA libraries ( Supplementary Figure S1A,B). The strand-specific RNA-seq libraries were prepared using the Illumina TruSeq Stranded mRNA Sample Preparation Kit (Illumina, San Diego, USA). The library was sequenced on the Illumina HiSeq X Ten System. We employed Trimmomatic [5] to remove low-quality reads, which resulted in approximately 89 million, 76 million, 72 million, and 56 million clean reads for the transcriptome and translatome of the early embryos and S2R+ cells, respectively. These reads were then mapped to the *Drosophila* genome (UCSC dm6) using HISAT2 [6]. The unique mapped reads ratio ranges from 94% to 85% and reads mapped to rRNA were less than 6% ( Supplementary Table S1), indicating the high quality of the four RNA-seq libraries. Using StringTie [7], 33,470 transcripts were assembled for four mapping sequencing libraries, which revealed an average of 1.9 transcribed transcripts and 1.8 translated transcripts per gene ( Supplementary Table S1), sugge sting the usage of transcript isoforms widely existed in both transcription and translation of *Drosophila* embryos.

To explore the divergence of the transcriptome during *Drosophila* development, we compared the transcriptome of the early embryos and S2R+ cells to identify genes with |log2(fold change)| ≥1, FPKM ≥1 in at least one condition, and adjusted *P* value ≤0.001. In total, we identified 2267 differentially expressed genes (DEGs) from 8815 genes. Among these DEGs, 2147 genes showed higher expression levels in the embryos, while 120 genes showed higher expression levels in S2R+ cells ( Figure 1A and Supplementary Figure S2A). To investigate the underlying functional mechanism, we performed enrichment analysis to identify DEG-enriched pathways ( Supplementary Table S2). Interestingly, the top 10 enriched pathways are related to morphogenesis and embryogenesis ( Figure 1B).

**Figure 1:**
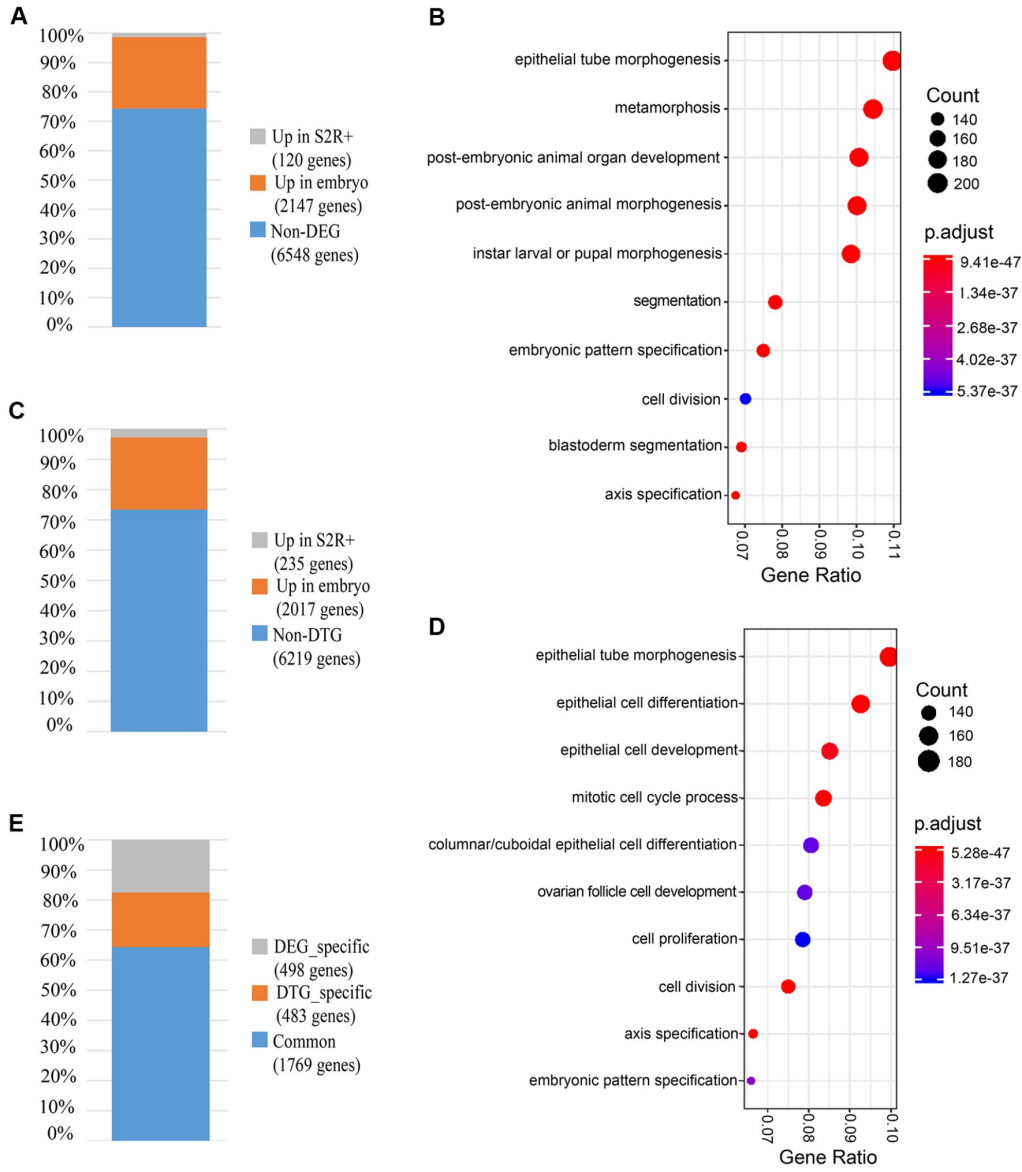
Figure 1 Analysis of the expression patterns of early embryos and S2R+ cells via the transcriptome and translatome (A) The number and proportion of DEGs between Drosophila early embryos and the S2R+ cell transcriptome. (B) Gene Ontology analysis of DEGs in the transcriptome. The top 10 DEG-enriched pathways are shown in a dot plot. (C) The number and proportion of differentially translated genes (DTGs) between Drosophila early embryos and the S2R+ cell translatome. (D) Gene Ontology analysis of DTGs in the translatome. The top 10 DTG-enriched pathways are shown in a dot plot. (E) Comparison of the DEGs between embryos and S2R+ cells and the DTGs between embryos and S2R+ cells.

To investigate translatome divergence during *Drosophila* development, we compared the translatome profiles between early embryos and S2R+ cells. With the pre-defined criteria (see Supplementary Methods), we identified 2252 differentially translated genes (DTGs) from 8471 expressed genes ( Figure 1C and Supplementary Figure S2B). Among these DTGs, 2017 genes showed higher expression levels in the embryos, and 235 genes showed higher expression levels in the S2R+ cells. The enriched pathways play an important role in embryo development ( Figure 1D and Supplementary Table S2). The comparison between DEGs in transcriptome and DTGs in translatome showed that a significant proportion (approximately 78%) of the DEGs in the transcriptome and the DTGs in the translatome consisted of 1769 shared genes ( Figure 1E). The pathways exhibiting the highest enrichment were found to be closely associated with embryogenesis and morphogenesis ( Supplementary Table S2). These results implied that those common genes in DEGs and DTGs are essential to maintain normal morphogenesis during embryogenesis.

Furthermore, we investigated the regulation of translational efficiency at different developmental stages in *Drosophila*. By comparing the mRNA abundance of cytosolic RNA and ribosome-associated RNA, whether the specific mRNA is preferentially translated could be determined. Referring to the definition of translational efficiency (TE) in previous research [8], we defined and calculated TEs for 8077 and 7497 genes in early embryos and S2R+ cells, respectively (see Supplementary Methods), and we identified 6758 common genes for further joint analysis. For the 6758 common genes, first, we normalized the TEs to range from 0 to 1 for comparison ( Figure 2A). We then found that 343 genes had differential TEs (the absolute difference in TEs between embryos and S2R+ cells are bigger than 0.5), of which 149 genes showed higher TE in embryos, 194 genes showed higher TE in S2R+ cells. The gene ontology analysis showed those genes enriched in cytoplasmic translation, axon guidance, and neuron projection guidance pathways ( Figure 2B and Supplementary Table S2). Inspection of differential TE genes revealed that the TE of the *comm2* gene in the early embryos was higher than that in S2R+ cells ( Figure 2C). *Comm2* plays an important role in the formation of the *Drosophila* embryonic central nervous system (CNS), which acts as a downstream regulator of *fra*, exerting control over axon guidance across the midline of the CNS. This suggested that translational regulation contributed to the development of the embryonic CNS.

**Figure 2:**
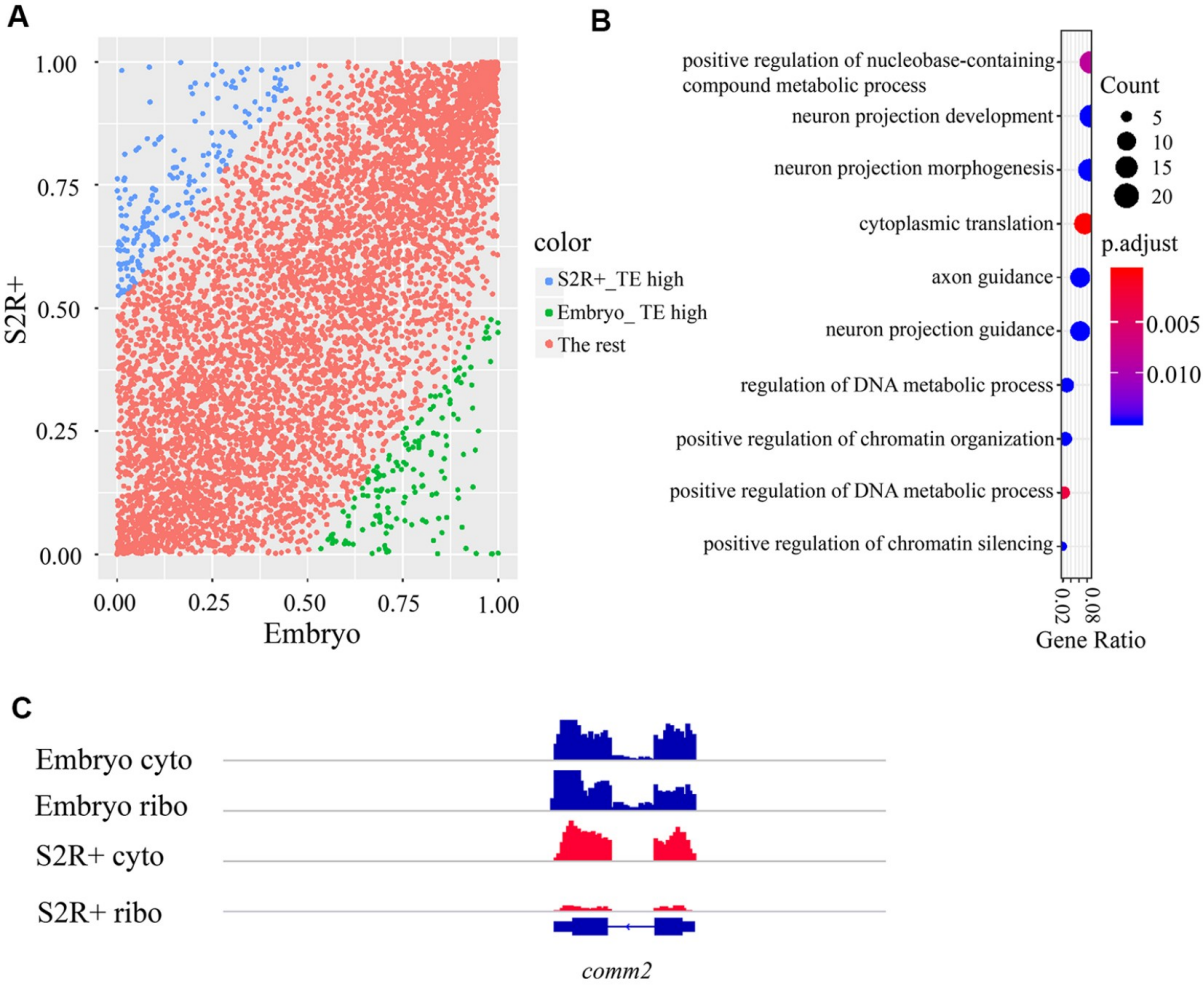
Figure 2 Analysis of TE on the gene level (A) Scatter plot for TE comparison between early embryos and S2R+ cells. The Y-axis corresponds to the TE value of genes in S2R+ cells, and the X-axis corresponds to the TE value of genes in early embryos. All TEs were normalized to the range of 0 to 1. The green dots represent genes with upregulated TEs in early embryos; the blue dots represent genes with upregulated TEs in S2R+ cells; and the red plots represent genes with absolute differences in TE between embryos and S2R+ cells smaller than 0.5. (B) Gene Ontology analysis of genes with differential TEs between early embryos and S2R+ cells. The top 10 DEG-enriched pathways are shown in a dot plot. (C) A snapshot of the IGV on the Drosophila comm2 gene. The tracks from top to bottom represent the following groups: embryo cytosolic RNA, embryo ribosome-associated RNA, S2R+ cytosolic RNA, and S2R+ ribosome-associated RNA. The translational efficiency of the comm2 gene was greater in early embryos than in S2R+ cells.

Selective mRNA isoform usage is widely observed in eukaryotes as a valuable method to increase the expression complexity of protein products from a limited number of genes [ 9, 10]. We calculated the selective usage of the isoform by ribosome (see Supplementary Methods) in the early embryos and S2R+ cells. There were 5634 and 4837 genes with at least 2 transcripts in the early embryo and S2R+ cell transcriptomes, respectively. In the early embryos, 32.1% of the genes used at least 2 transcripts in the transcriptome ( Supplementary Figure S3A); in contrast, the percentage of genes still using ≥2 transcripts decreased significantly in the translatome (29.6%, *P* value=5.661×10 ^–7^ by proportion test). In S2R+ cells, 27.5% of the genes used at least 2 transcripts in transcription; in contrast, the percentage of genes still using ≥2 transcripts decreased significantly in the translatome (24.4%, *P* value=2.193×10 ^–11^ according to the proportion test). Using the slightly modified chi-square statistical framework (see Supplementary Methods), we identified 3986 and 3961 genes that underwent significant selective usage of isoforms in early embryos and S2R+ cells, respectively ( Supplementary Table S3). Surprisingly, 2931 (approximately 74%) of these genes underwent significant translational selection in both early embryos and S2R+ cells ( Supplementary Figure S3B). For genes with multiple transcribed isoforms, we defined a *Diff_CP* score to measure the selection power of ribosomes on each isoform of one gene (see Supplementary Methods). Upon comparing the disparities in mRNA structural elements between the maximally positively selected transcript and the maximally negatively selected transcript in genes subjected to translational selection (see Supplementary Methods), we observed a strong correlation between the translational preference and the 3′UTR and 5′UTR in both early embryos (approximately 68%) and S2R+ cells (approximately 66%) ( Supplementary Figure S3C). Since the 5′ UTR and 3′UTR affect translation efficiency through mechanisms such as the interaction of cis-acting elements and trans-acting factors, the above results suggested that the gain or loss of sequence elements related to specific transcript isoforms could be an important contributing factor to translational regulation in *Drosophila* embryogenesis.

We compared the number of isoforms per gene for the expressed genes between the transcriptome and translatome data ( Supplementary Figure S3D,E). In the early embryos, approximately 58% of the expressed genes exhibited the same number of transcripts in the transcriptome ( Supplementary Figure S3D). In comparison, the percentage for S2R+ cells increased to 87% ( *P*<2.2×10 ^–16^), indicating that there was more post-transcriptional regulation in the early embryos than in S2R+ cells ( Supplementary Figure S3E). We defined the dominant transcript ( Supplementary Methods) as the isoform that was expressed at a considerably higher level than the other isoforms of one gene. In the early embryos, 65% of the dominant transcripts remained unchanged between cytosolic RNA and ribosome-associated RNA, and 59% of the dominant transcripts remained unchanged in S2R+ cells between cytosolic RNA and ribosome-associated RNA ( Supplementary Table S4). The switch of dominant transcripts in early embryos and S2R+ cells suggested that ribosome selection on specific isoforms is ubiquitous in *Drosophila* embryogenesis, which underscores the need for further investigation of the mechanisms leading to this switch.

The comprehensive investigation of the transcriptome and translatome of early embryos and S2R+ cells offered us insights into the dynamics of transcriptional and translational regulation during *Drosophila* embryogenesis. We identified 2267 DEGs and 2252 DTGs, and the common genes were enriched in essential pathways related to embryogenesis and morphogenesis, including axis specification, epithelial tube morphogenesis, epithelial cell development, and differentiation. Axis specification, the first step specifying each region of early embryos, is crucial for establishing embryonic axes, and axial information is then used to generate regional differences within the embryo. The early stages of *Drosophila* embryogenesis involve a series of morphogenetic events, among which extensive reorganization of the embryonic epithelium is a necessary process. These findings indicate that the DEGs and DTGs identified here are valuable for future studies of embryonic development.

In summary, we combined transcriptome and translatome data to study the regulation of gene expression during *Drosophila* embryogenesis. By analyzing early embryos and S2R+ cells, we identified 2267 DEGs and 2252 DTGs, with a significant proportion of shared genes associated with essential pathways involved in morphogenesis and embryonic development. We calculated TEs for the 6758 common genes and identified 343 genes with significant differential expression patterns, of which 149 exhibited higher TE in embryos and 194 in S2R+ cells. Our research highlighted TE regulation at different *Drosophila* developmental stages, revealing genes enriched in pathways crucial for embryo development. In addition, we identified 3986 and 3961 genes that underwent significant selective usage of mRNA isoforms by ribosomes, with a strong correlation between translational preference and the 3′ and 5′ UTR regions, respectively. Overall, the integration of transcriptome and translatome data provides a comprehensive understanding of gene expression dynamics during *Drosophila* embryogenesis, with valuable knowledge contributing to developmental biology.

## Supporting information

Table_S1

Table_S4

Table_S3

103Supplementary_materials

Table_S2

## Abbreviations

The datasets generated in this study can be found in the NCBI Sequence Read Archive (SRA) under accession number PRJNA990480:
http://www.ncbi.nlm.nih.gov/bioproject/990480.

## Supplementary Data

Supplementary data is available at *Acta Biochimica et Biophysica Sinica* online.
