# Supplementary material for: Co-profiling of translatome and transcriptome reveals the regulation of dynamic gene expression during Drosophila embryogenesis : Co-profiling of translatome and transcriptome during Drosophila embryogenesis
Source: Acta Biochim Biophys Sin (Shanghai). 2024 Sep 2;56(11):1711–5. doi: 10.3724/abbs.2024146 (PMC11693868; doi:10.3724/abbs.2024146)
Supplement: 103Supplementary_materials [file 103Supplementary_materials.pdf]

Supplementary Materials

Supplementary Methods

Culture of S2R+ cells

The S2R+ cell line was kindly provided by Prof. Nan Liu from the Shanghai Institute of Organic Chemistry, Chinese Academy of Sciences. S2R+ cells were grown in Schneiders medium (Invitrogen, Carlsbad, USA) supplemented with 10% heat-inactivated FBS (Gibco, Carlsbad, USA) at 25 ℃ [1].

Collection of early embryos

Early (0–4 h) embryos were collected from egg-laying dishes, followed by dechorionation using a 3–5 min treatment with 50% bleach [2]. Subsequently, the early embryos were thoroughly washed with 1 × PBS buffer.

Ribosome and cytosolic RNA preparation for S2R+ cells and early embryos

The methods of ribosome and cytosolic RNA preparation used were those described by Hongmei Li [2]. S2R+ cells were treated with cycloheximide at 100 μg/mL for 15 min at room temperature before harvesting. The cells were pelleted, immediately washed with PBS (with 100 μg/mL cycloheximide), resuspended in a mild ribosome lysis buffer (50 mM Tris-HCl, pH 7.4, 150 mM NaCl, 10 mM $MgCl_2$, 1 mM DTT, 1% Triton X-100, 100 μg/ml cycloheximide, and 0.5 unit/mL RNasin) and incubated for 15 min on ice. Dechorionated early embryos were incubated with 100 μg/mL cycloheximide for 5 min on ice, followed by homogenization in 100 μL of ribosome lysis buffer using a plastic pestle. After the lysis of S2R+ cells and dechorionated embryos, the resulting mixture was centrifuged at 16,000 *g* for 15 min at 4 ℃ to remove the nuclei and whole cells. The resulting cytosolic supernatant was then loaded onto 10% to 50% continuous sucrose density gradients with the lysis buffer (50 mM Tris-HCl, pH 7.4, 150 mM NaCl, 10 mM $MgCl_2$, 1 mM DTT) and subjected to ultracentrifugation at 210,000 *g* for 2.5 h at 4 ℃ using a SW41 rotor (Beckman, Pasadena, USA). The absorbance of each layer in the sucrose density gradient was measured at 254 nm using the Piston Gradient Fractionator (BioComp Instruments, Fredericton, Canada). RNA components and the number of ribosomes were determined based on distinct peaks observed in the polysome profiling. Considering the importance of monosome for the translation [3,4], ribosome material was collected by pooling both the 80S (monosome) and polysome peaks identified in the profile. The ribosome-associated RNA and cytosolic RNA were extracted by adding an equal volume of Trizol reagent (Invitrogen) to the

ribosome material and cytosolic supernatant, respectively, followed by chloroform extraction and ethanol precipitation.

Strand-specific RNA-seq library construction

The RNA concentration was quantified by Nanodrop2000 (Thermo Scientific, Waltham, USA) and the RNA quality was detected with Agilent Bioanalyzer 2100. The method to purify poly-adenylated RNA of ribosome-associated RNA and cytosolic RNA were optimized using the RiboMinus Eukaryote Kit for RNA-Seq (#A10837-08; Ambion, Austin, USA) to delete ribosomal RNAs (rRNAs) and Dynabeads oligo (dT)25 (#61002; Life Technologies, Carlsbad, USA) purification to select RNAs with poly-adenylated tails. The strand-specific RNA-seq library of the ribosome-associated poly-adenylated RNA was prepared using the Illumina TruSeq Stranded mRNA Sample Preparation Kit (cat# RS-122-2101; Illumina, San Diego, USA). The library was sequenced on the Illumina HiSeq X Ten System. These sequence data have been submitted to the NCBI SRA databases under accession number SRP446994.

RNA-seq analysis

The low-quality reads were removed with Trimmomatic [5]. Then, the reads were aligned to *Drosophila* melanogaster transcriptome (dm6) using HISAT2 V.2.0.5 [6] specifying the "-G" parameter and "--no-soft-clip" parameter while the other parameters set to default. Only the uniquely aligned and concordant read pairs were used for further analysis. We used StringTie V1.3.3 [7] to assemble transcripts and to quantify Transcript-level FPKM values using default parameters with *Drosophila* melanogaster BDGP6.90.gtf annotation file. The gene-level differential expression analysis was conducted using the DEGseq R package [8] with FPKM value. The criteria for differential expression are Log2(Fold change) >1 or Log2(Fold change) <-1 with adjusted *P*-value <0.05 (using Benjamini–Hochberg method). The GO analysis and visualization were conducted using clusterProfiler R package [9] . RNA-seq based plots and other figures were prepared with Excel and IGV [10].

Analysis of the translational efficiency of the early embryos and S2R+ cells

To evaluate the translational efficiency of each gene, we defined the ribosome binding efficiency as translational efficiency (TE):

$$Translational\_Efficiency = \frac{ribo\_FPKM}{cyto\_FPKM}$$

ribo_FPKM denotes the FPKM value of one gene in ribosome-associated RNA, and cyto_FPKM denotes the corresponding FPKM value of one gene in cytosolic RNA. Before the calculation of ribosome binding efficiency, low expression abundance genes were filtered with the criteria below: (1) Genes with FPKM >1 in both cytosolic and ribosome-associated RNA data were maintained to calculate ribosome binding efficiency. (2) Only genes with FPKM >1 in both S2R+ and early embryos were compared for the difference in ribosome binding efficiency between the two cell types.

Calculation of selection power of transcript and probability of significant translational selections

With the criterion of FPKM >1, we filtered out the genes with low expression in cytosolic RNA and ribosome-associated RNA data and used the remaining genes for downstream analysis. To measure the selection power during the process of translation, we first defined the relative expression value RP_i for each transcript of a given gene as follows:

$$RP_i = \frac{T_i}{\sum_{j=1}^{n} T_i}$$

RP_i is the relative expression value of the ith transcript; T_i is the FPKM value of the ith transcript;$\sum_{j=1}^{n} T_i$ is the sum of all transcripts' expression values of the gene. Then, we defined the *Diff_CP* values as follows to measure the difference between RP_i in cytosolic RNA and ribosome-associated RNA.

$$Diff\_CP_i = cyto\_RP_i - \text{ribo}\_RP_i$$

$cyto\_RP_i$ is the relative expression value of the ith transcript in cytosolic RNA, and $ribo\_RP_i$ is the relative expression value of the ith transcript in ribosome-associated RNA. The value range of $Diff\_CP_i$ is [-1,1]. Obviously, a smaller $Diff\_CP_i$ value means a stronger selection power on the ith transcript by ribosome. We then defined the *Positive_tran* and *Negative_tran* as follows:

$$Positive\_tran = min\{Diff\_CP_1, Diff\_CP_2, \ldots, Diff\_CP_n\}, i \in \{1, \ldots, n\}$$

$$Negative\_tran = max\{Diff\_CP_1, Diff\_CP_2, \ldots, Diff\_CP_n\}, j \in \{1, \ldots, n\}$$

n is the number of transcripts of the gene. *Positive_tran* corresponds to the maximumly positively selected transcript of the gene, and *Negative_tran* corresponds to the maximumly negatively selected transcript. Finally, we defined the selection power of ribosome on the gene as follows:

$$Selection_{power} = \frac{|Positive_{tran}| + |Negative_{tran}|}{2}$$

A bigger value of Selection_power means a stronger overall selection power on specific transcripts by ribosome.

To determine whether a gene underwent significant selective translation, two prerequisites had to be met in the first place: (1) Genes had at least 2 expressed transcript isoforms; (2) The FPKM value of at least one transcript was greater than 1. For those genes that satisfy the criteria above, we designed a statistical method based on a chi-squared test to calculate the probability of selective usage of the transcript: (1) Use the FPKM in cyto and ribo count data to construct the contingency table; (2) use the *chi2_contingency* function in python *scipy.stats* module to calculate the original *P*-value (3) use the B-H correction method to obtain the false discovery rate (FDR). Only the genes with FDR <0.05 were considered to undergo significant selective usage of transcript during translation.

Identification of dominant transcript

If a gene had at least 2 expressed transcript isoforms and the FPKM value of at least one transcript was greater than 1, we defined its dominant transcript as the transcript isoform expressed at a level higher than its other isoforms'. The dominant transcript is determined as follows:

$$dominant\_tran = which.max\{ trans_1, trans_2, \dots, trans_i\}, i \in \{1, \dots, n\}$$

$trans_i$ is the expression value of the ith transcript, *dominant_tran* is the transcript with maximum expression value in all transcripts of a gene.

Supplementary Figures

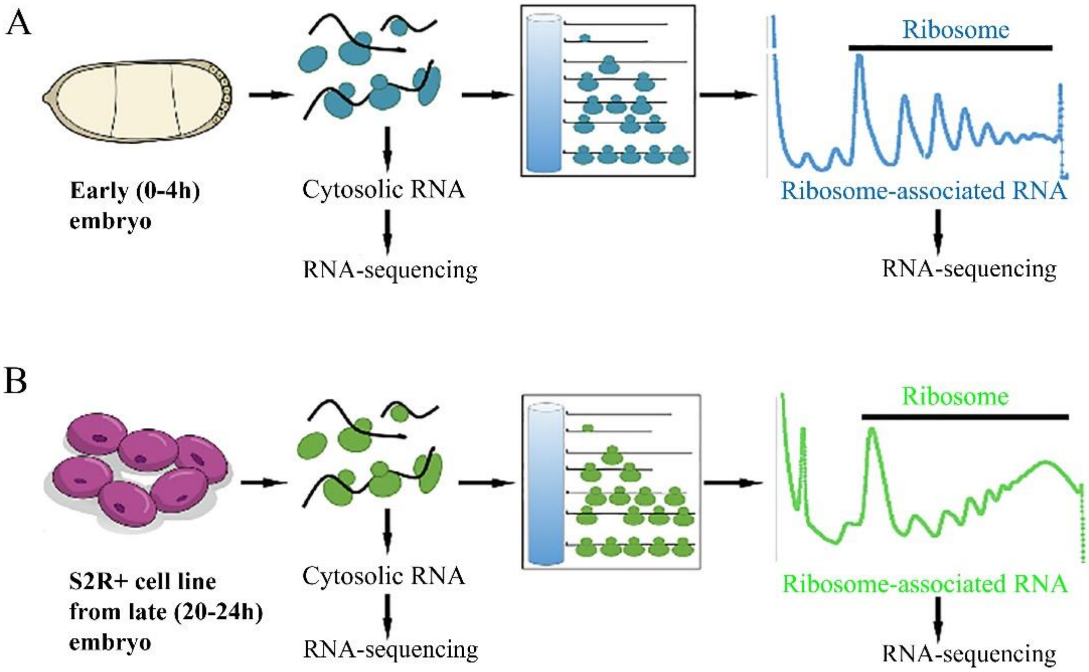

Supplementary Figure S1. Schematic diagram of the workflow Schematic diagram illustrating the process of sample preparation and sequencing of cytosolic RNA and ribosome-associated RNA from *Drosophila* early (0-4 h) embryos (A) and S2R+ cells (from late (20-24 h) embryo) (B).

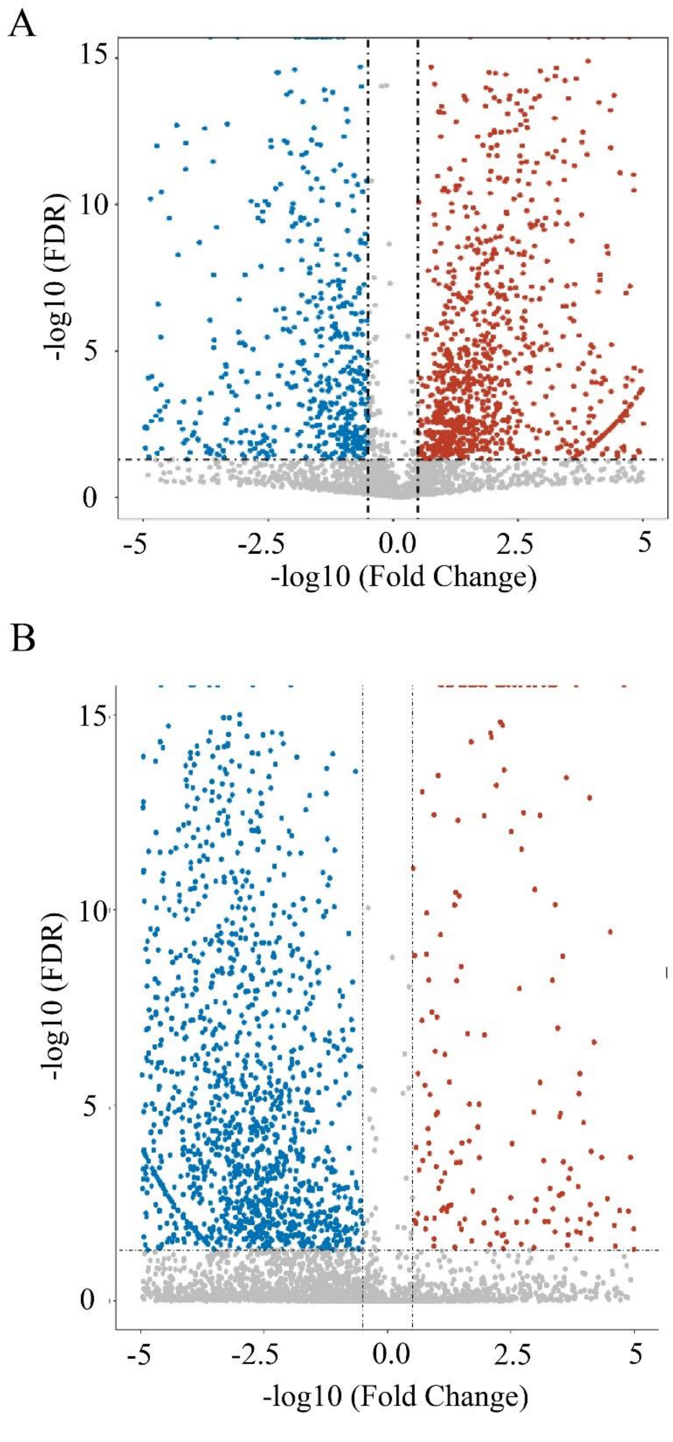

Supplementary Figure S2. DEGs and DTGs between early embryos and S2R+ cells Volcano plot displaying DEGs between early embryos and S2R+ cells. The red dots represent the expression value of upregulated genes; the blue dots represent the expression value of downregulated genes (A). Volcano plot displaying DTGs between early embryos and S2R+ cells (B).

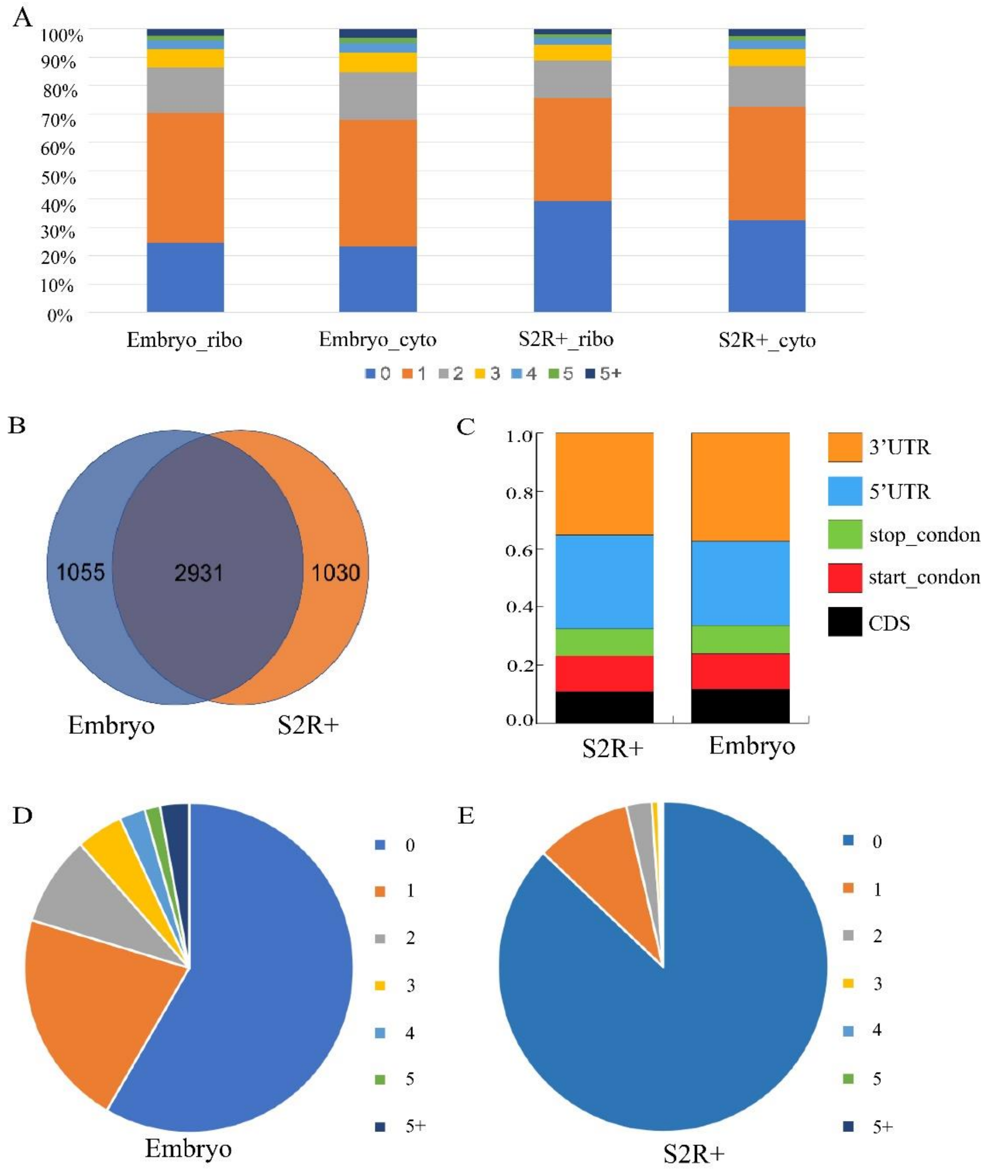

Supplementary Figure S3. Analysis of selective usage on isoform level (A) Statistics of isoform usage for each gene in different samples. The number represents how many isoforms are transcribed for one gene. (B) Comparison of genes with significant selective usage of isoforms in early embryos and S2R+ cells. The overlap represents the number of common genes that occurred in significant selective usage of isoforms. (C) Statistics of mRNA structural difference between max positively and max negatively selected isoforms in early embryos and S2R+ cells. (D) Statistics of difference in isoform usage for each gene between cytosolic RNA and ribosome-associated RNA of early embryos. 0 means one gene using the same number of isoforms in cytosolic RNA and ribosome-associated RNA. (E) Statistics of difference in isoform usage for each gene between cytosolic RNA and ribosome-associated RNA of S2R+ cells.

Supplementary Tables

Supplementary Table S1. Statistics of reads (worksheet 1) and Transcripts per gene (worksheet 2) In the table, “Ribo” represents ribosome-associated RNA and “cyto” represents cytosolic RNA.

Supplementary Table S2. Gene Ontology (GO) enrichment was analyzed in different groups GO analysis of DEGs (worksheet 1), GO analysis of DTGs (worksheet 2), GO analysis of High TE genes (worksheet 3), GO analysis of the common portion between DEGs and DTGs (worksheet 4), GO analysis of conserved selective genes (worksheet 5).

Supplementary Table S3. Selected usage of isoforms in S2R+ and early embryos

Supplementary Table S4. Dominant transcripts in each group Dominant transcripts in Embryo_Cyto (worksheet 1); Dominant transcripts in Embryo_Ribo (worksheet 2); Dominant transcripts in S2R+_Cyto (worksheet 3); Dominant transcripts in S2R+_Ribo (worksheet 4). “Ribo” represents the ribosome-associated RNA and “Cyto” represents cytosolic RNA.

References

1. Wang Q, Sun Q, Czajkowsky DM, Shao Z. Sub-kb Hi-C in D. melanogaster reveals conserved characteristics of TADs between insect and mammalian cells. Nat Commun 2018, 9: 188.
2. Li H, Hu C, Bai L, Li H, Li M, Zhao X, et al. Ultra-deep sequencing of ribosome-associated poly-adenylated RNA in early Drosophila embryos reveals hundreds of conserved translated sORFs. DNA Res 2016, 23: 571-580.
3. Biever A, Glock C, Tushev G, Ciirdaeva E, Dalmay T, Langer JD, et al. Monosomes actively translate synaptic mRNAs in neuronal processes. Science 2020, 367: eaay4991
4. Heyer Erin E, Moore Melissa J. Redefining the translational status of 80S monosomes. Cell 2016, 164: 757-769.
5. Bolger AM, Lohse M, Usadel B. Trimmomatic: a flexible trimmer for Illumina sequence data. Bioinformatics 2014, 30: 2114-2120.
6. Kim D, Paggi JM, Park C, Bennett C, Salzberg SL. Graph-based genome alignment and genotyping with HISAT2 and HISAT-genotype. Nat Biotech 2019, 37: 907-915.
7. Pertea M, Pertea GM, Antonescu CM, Chang T-C, Mendell JT, Salzberg SL. StringTie enables improved reconstruction of a transcriptome from RNA-seq reads. Nat Biotech 2015, 33: 290-295.
8. Pertea M, Kim D, Pertea GM, Leek JT, Salzberg SL. Transcript-level expression analysis of RNA-seq experiments with HISAT, StringTie and Ballgown. Nat Protoc. 2016;11(9):1650-67.
9. Yu G, Wang LG, Han Y, He QY. clusterProfiler: an R package for comparing biological themes among gene clusters. Omics 2012, 16: 284-287.
10. Thorvaldsdóttir H, Robinson JT, Mesirov JP. Integrative Genomics Viewer (IGV): high-performance genomics data visualization and exploration. Brief Bioinform 2013, 14: 178-192.
